# LncRNAs Expression in Preeclampsia Placenta Reveals the Potential Role of LncRNAs Contributing to Preeclampsia Pathogenesis

**DOI:** 10.1371/journal.pone.0081437

**Published:** 2013-11-28

**Authors:** Xiaoju He, Yinyan He, Binrong Xi, Jiusheng Zheng, Xiaoming Zeng, Qinhua Cai, Yu OuYang, Chen Wang, Xiaofei Zhou, Huiying Huang, Wei Deng, Siming Xin, Qixiang Huang, Huai Liu

**Affiliations:** 1 Department of Obstetrics, Jiangxi Maternal and Child Health Hospital, Nanchang, China; 2 Department of Obstetrics & Gynecology, Shanghai First People's Hospital, School of Medicine, Shanghai Jiao Tong University, Shanghai, China; 3 School of Medicine, Nanchang University, Nanchang, China; VU University Medical Center, Netherlands

## Abstract

**Background:**

Long non-coding RNAs (lncRNAs) are an important class of pervasive genes involved in a variety of biological functions. They are aberrantly expressed in many types of diseases. In this study, we aimed to investigate the lncRNA profiles in preeclampsia. Preeclampsia has been observed in patients with molar pregnancy where a fetus is absent, which demonstrate that the placenta is sufficient to cause this condition. Thus, we analyzed the lncRNA profiles in preeclampsia placentas.

**Methodology/Principal Findings:**

In this study, we described the lncRNA profiles in six preeclampsia placentas (T) and five normal pregnancy placentas (N) using microarray. With abundant and varied probes accounting for 33,045 LncRNAs in our microarray, 28,443 lncRNAs that were expressed at a specific level were detected. From the data, we found 738 lncRNAs that were differentially expressed (≥1.5-fold-change) among preeclampsia placentas compared with controls. Coding-non-coding gene co-expression networks (CNC network) were constructed based on the correlation analysis between the differentially expressed lncRNAs and mRNAs. According to the CNC network and GO analysis of differentially expressed lncRNAs/mRNAs, we selected three lncRNAs to analyze the relationship between lncRNAs and preeclampsia. LOC391533, LOC284100, and CEACAMP8 were evaluated using qPCR in 40 preeclampsia placentas and 40 controls. These results revealed that three lncRNAs were aberrantly expressed in preeclampsia placentas compared with controls.

**Conclusions/Significance:**

Our study is the first study to determine the genome-wide lncRNAs expression patterns in preeclampsia placenta using microarray. These results revealed that clusters of lncRNAs were aberrantly expressed in preeclampsia placenta compared with controls, which indicated that lncRNAs differentially expressed in preeclampsia placenta might play a partial or key role in preeclampsia development. Misregulation of LOC391533, LOC284100, and CEACAMP8 might contribute to the mechanism underlying preeclampsia. Taken together, this study may provide potential targets for the future treatment of preeclampsia and novel insights into preeclampsia biology.

## Introduction

 Preeclampsia is characterized by hypertension and de novo proteinuria after 20 weeks of pregnancy. It is the leading cause of perinatal morbidity and mortality worldwide, and to date, the only means of treating this disease is by inducing delivery. Preeclampsia affects 3-5% of all pregnancies and is estimated to result in 60,000 maternal deaths annually worldwide [1].

 The origin of the disease is the placenta, but its sequelae affects multiple organ systems. Endothelial dysfunction is the common denominator of the clinical symptoms. This theory may also underlie the origins of hypertension, proteinuria, edema and other symptoms as well [2]. Basic research has shown that genetic events play a major role in the development of preeclampsia, particularly, the gene of fms-like tyrosine kinase 1(Flt-1), which might be one of the important genetic events in preeclampsia. Recent studies have shown that the major phenotypes of preeclampsia, such as hypertension and proteinuria, are due to soluble sFlt-1(sFlt-1). sFlt-1 acts to neutralize the pro-angiogenic proteins, vascular endothelial growth factor (VEGF) and placental growth factor (PlGF)[3], which is also known as sVEGFR-1. 

 Recently, genetic studies have focused on non-coding RNAs. These abundant transcriptomes are regarded as “transcriptional noise.” However, over the past decade, many studies have reported that these non-coding RNAs have a series of important regulatory potential both in transcription and post transcription [4]. LncRNAs are defined as non-coding RNAs that are longer than 200 nucleotides in length. Increasing evidence indicates that lncRNAs exhibit important roles during both normal development and disease. Misregulation of lncRNAs has been shown to be associated with many human diseases [5]. Large-scale analysis of full-length cDNA sequences have detected a large number of long non-coding RNAs in human, mouse, and fly. These lncRNAs have been shown to exhibit key roles in imprinting control, cell differentiation, immune responses, human diseases and other biological processes [6,7]. Because preeclampsia is a disease during pregnancy, these lncRNAs are expressed in a temporal and site-specific fashion, which potentially regulates its functions during the development of the disease. However, the expression of lncRNAs and their biological functions in preeclampsia still remain unknown.

 In this study, we examined the lncRNA expression profiles of six cases of preeclampsia placenta compared with five-matched control samples, where several of the differentially expressed lncRNAs were evaluated using qPCR in a total of eighty placenta tissues. Our results demonstrated that lncRNA expression profiles may provide new molecular biomarkers or a new basis for the diagnosis and treatment of preeclampsia.

## Results

### Overview of lncRNA Profiles

 Based on the lncRNAs expression profiles (Table S3), differentially expressed lncRNAs can be found between the preeclampsia (T) and normal samples (N). The expression profiles of lncRNAs were shown by calculating the log-fold change (T/N). We determined that 738 differentially expressed human lncRNAs in RefSeq_NR, UCSC_knowngene, Ensembl, H-invDB, Fantom, Fantom_stringent, NRED, RNAdb, misc_lncRNA, UCR and lncRNA in six preeclampsia patients.

 NR_027457 (Log_2_ Fold change T/N=4.8407316) was the most significantly up-regulated lncRNA while G36948 (Log_2_ Fold change T/N= -4.713349) was the most significantly down-regulated lncRNA (Table 1). There were 259 up-regulated lncRNAs and 479 down-regulated lncRNAs identified (Table S1). 

**Table 1:** A collection of deregulated lncRNAs detected using microarray.

up-regulated		down-regulated	
lncRNAs	Log_2_ Fold change (T/N)	LncRNAs	Log_2_ Fold change (T/N)
NR_027457	4.8407316	G36948	-4.713349
NR_024178	3.7026873	NR_029420	-3.944745
ENST00000508010	3.6478987	ENST00000462801	-3.5958498
nc-HOXD4-22-	3.6392443	ENST00000420346	-3.4068155
BC030099	3.1557207	uc001yen.1	-3.3312943
AF037219	3.0707293	ENST00000452363	-3.1808643
ENST00000426615	3.0671024	ENST00000514942	-3.1543686
uc001xoi.1	3.0389638	NR_024015	-3.0237393
uc002 µkl.1	3.0248022	AK002210	-3.022271
AF085938	2.9179204	NR_026643	-2.9749532
ENST00000453697	2.8973458	AL049277	-2.8766768
G43016	2.8749487	ENST00000443801	-2.8124766
ENST00000504200	2.7703688	CR619533	-2.7963133
AK055151	2.7125444	chr5:32328662-32332385+	-2.7114208
uc002ckp.1	2.6354249	nc-HOXC10-127+	-2.701504
NR_026824	2.624768	ENST00000416513	-2.68905
ENST00000508921	2.6117814	NR_027435	-2.6723933
ENST00000507681	2.5811958	ENST00000402635	-2.653753
CR591190	2.57254	AY034469	-2.6396053
ENST00000441562	2.5398467	ENST00000453648	-2.634475

### Overview of mRNA Profiles

 Up to 18,063 coding transcripts could be detected in the placenta samples using 30,215 coding transcript probes (Table S5). Among the two groups of placenta samples, 225 mRNAs were up-regulated in preeclampsia compared with the matched normal tissues, while 211 mRNAs were down-regulated (Table S6). GO and Pathway analyses showed that the differentially expressed mRNAs were related with pregnancy and were involved in lipid metabolism and the regulation of the type 2 immune response. These results supported the idea that preeclampsia is a metabolic and immune disease of pregnancy (Table S7).

### Construction of the Coding-non-coding Gene Coexpression Network

**Figure 1:**
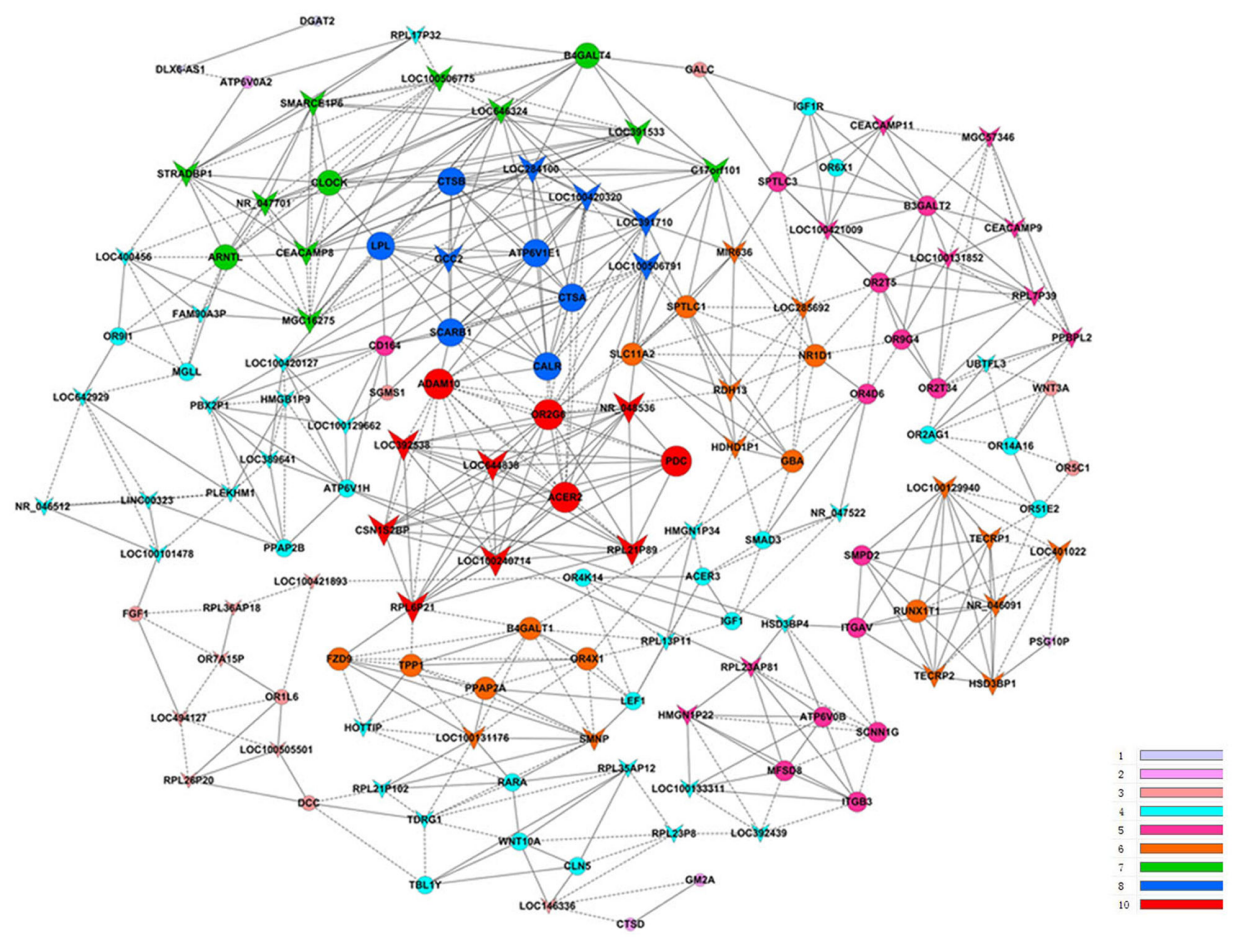
Coding-non-coding gene co-expression network.

 The coding-non-coding gene co-expression network (CNC network) was constructed based on the correlation analysis between differentially expressed lncRNAs and mRNAs. LncRNAs and mRNAs with Pearson correlation coefficients above 0.95 were selected for the network using the cytoscape program. Among the co-expression network, 68 lncRNAs and 73 mRNAs comprise the CNC network node. In addition, 141 network nodes were associated with 555 network pairs of co-expressing lncRNAs and mRNAs, and most of these pairs were presented as a positive correlation. The CNC networks indicated that one mRNA was correlated with one to ten lncRNAs (Figure 1). 

In figure, the circular nodes represent the mRNA, V on behalf of lncRNA. The solid lines represent a positive correlation, and the dashed lines indicate a negative correlation. The same color nodes represent co-expressed genes with a similar capacity chart. The node size indicates that the expression of the gene, and the lines represent the gene co-expression relationship of the gene. K-core indicates the gene expression.

 According to the GO-Pathway analysis of differentially expressed lncRNAs/mRNAs (Table S4), we selected three lncRNAs: LOC391533, LOC284100, and CEACAMP8. At the top of the coding-non-coding gene co-expression network in Figure 1, three lncRNAs and their associated lncRNAs and mRNAs are identified, with most of the pairs presented as a positive correlation. LOC391533 is fms-like tyrosine kinase 1 (vascular endothelial growth factor/vascular permeability factor receptor) pseudogene (FLT1P1). Recent studies have revealed that the major phenotypes of preeclampsia, such as hypertension and proteinuria, are due to sFlt-1. LOC284100 and CEACAMP8 are associated with LPL mRNA expression, while hyperlipidemia is one common complication of preeclampsia. The CNC network implicates the inter-regulation of lncRNAs and mRNAs in preeclampsia. 

### Real-time Quantitative PCR Validation

**Figure 2:**
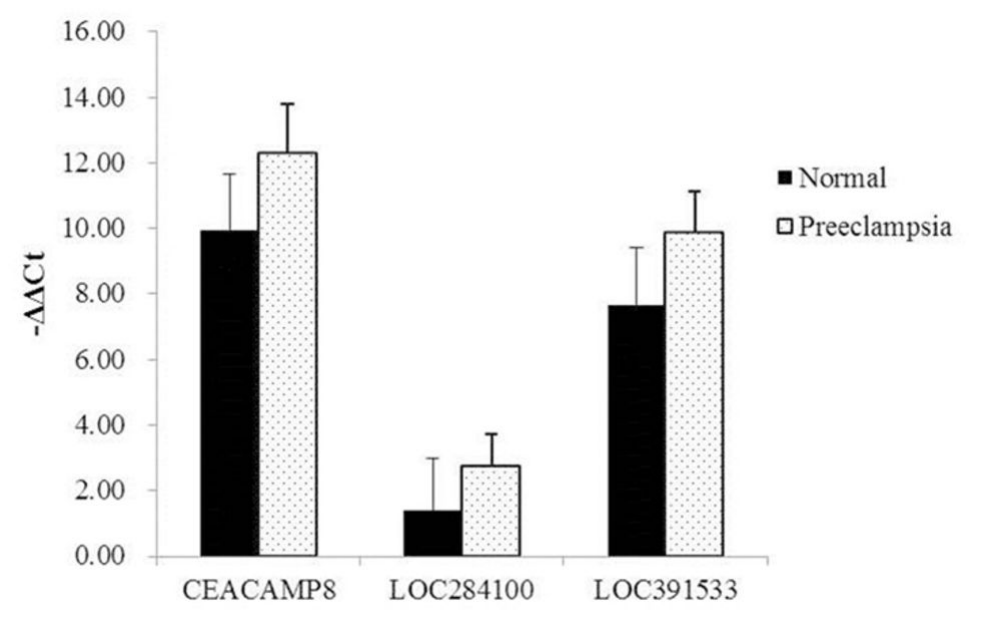
Delta-Ct values of real-time qPCR. Three lncRNAs were confirmed using qPCR in 40 preeclampsia placenta and 40 control placenta samples (p<0.05 for each lncRNAs). The bars indicate the standard deviation.

 According to the CNC network and the GO analysis of LncRNA/mRNA expression profiling, we examined the expression of three lncRNAs (LOC391533, LOC284100, CEACAMP8) in 40 preeclampsia placenta tissues and 40-matched control placenta tissues using qPCR (p<0.05 for each lncRNAs). These data indicated that LOC391533, LOC284100, and CEACAMP8 were up-regulated in preeclampsia samples compared with NT samples (Figure 2, Table S8).

## Discussion

 Preeclampsia is a systemic complication of pregnancy, which presents with hypertension, proteinuria and pathologic edema. Its laboratory results consist of elevated uric acid levels, hypoproteinemia, hyperlipidemia, and serum transaminase concentrations that are at least two-fold greater than normal samples. To date, the only means of treating this disease is the induction of delivery; thus, the placenta is the origin of this disease.

 The pathogenic mechanisms underlying preeclampsia remain to be elucidated. However, immune maladaptation, inadequate placental development and trophoblast invasion, placental ischemia, oxidative stress and thrombosis are all thought to represent key factors in the development of the disease. Furthermore, these components have genetic factors that may be involved in these pathogenic changes [8,9]. Because preeclampsia is a phenotypically complex disease, it is unlikely that any single gene will play a dominant role in its development [10,11]. Increasing evidence confirmed that LncRNA can regulate related protein coding genes. Moreover, LncRNAs regulate gene expression at the epigenetic, transcription, and post-transcription level [12].

 Previously, there have been no reports describing the expression profiles of lncRNAs in preeclampsia and there have been no studies on the association of lncRNA expression with the clinical characteristics and outcomes of preeclampsia. We analyzed six preeclampsia and five normal pregnancy placentas using microarray. We selected three lncRNAs for validation using qPCR in 80 samples. Using abundant and varied probes accounting for 33,045 lncRNAs and 30,215 coding transcripts in the microarray, a large number of lncRNAs may be quantitatively determined. Comprehensive in-depth analysis of the expression profiles of lncRNAs in preeclampsia was performed to investigate the role of lncRNAs in the development and progression of preeclampsia. Based on microarray data, we found that 28,443 lncRNAs were expressed, while 738 lncRNAs were differently expressed. Moreover, 259 lncRNAs were up-regulated and 479 lncRNAs were down-regulated, most of which have not been functionally characterized. These findings may likely provide a potential strategy to distinguish between preeclampsia placenta tissue and normal placenta tissue. Although noncoding RNAs in body fluid, such as serum and urine, have been identified as potential biomarkers [13], these samples were all term placenta. Thus, it was too early to utilize the three LncRNAs as potential biomarkers in preeclampsia based on the present data, which would be beneficial for the identification of novel molecular markers in preeclampsia. Most of the lncRNAs have a distinct spatial and temporal specificity in organismal differentiation and development. One study examining 1300 mouse lncRNAs demonstrated that in different parts of the brain, lncRNAs exhibit different expression patterns [14]. Moreover, signatures of lncRNA expression have been described in prostate carcinoma and hepatic tumors [15]. In the development of preeclampsia, there may be different expression patterns of lncRNAs and the differentially expressed lncRNAs may exert specific cellular functions in preeclampsia. These lncRNAs might also be involved in the development and progression of preeclampsia and may provide novel insight into the molecular basis of this disease.

 Based on the CNC network and the GO analysis of differentially expressed lncRNAs/mRNAs, three lncRNAs (LOC391533, LOC284100, CEACAMP8) were selected to validate its consistency. The expression of these three lncRNAs was further evaluated using qPCR in 80 placenta samples. LncRNA LOC391533 is a 1,434 bp intragenic lncRNA transcript from the LOC391533 gene located on Chromosome 3p21.31. This gene is fms-like tyrosine kinase 1 pseudogene 1 (FLT1P1) (http://www.ncbi.nlm.nih.gov/gene/?term=391533), which is similar to the FLT1 gene. The FLT1 gene is located on Chromosome 13q12. FLT1 encodes a member of the VEGFR family. This protein plays an important role in angiogenesis and vasculogenesis and consists of different isoforms, including a full-length transmembrane receptor isoform and a shortened, soluble isoforms. Soluble isoforms (sFlt-1) are associated with the onset of preeclampsia. Similar to functional genes, some pseudogenes also exhibit developmentally regulated expression patterns. For example, the expression of the high mobility group A1 pseudogene (HMGA1-p) can trigger the destabilization of HMGA1 mRNA. Because the HMGA1 protein participates in the regulation of the insulin receptor (INSR), the expression of HMGA1-p plays an important role in the onset of type 2 diabetes [16]. Furthermore, in preeclampsia development, the differential expression of LOC391533 may execute a specific function in preeclampsia. Although the regulatory functions of pseudogenes appear to be striking, functional pseudogene studies are still in the early stages. Thus, how LOC391533 interacts with Flt-1 requires further study.

 LncRNA LOC284100 is a 41,791 bp intragenic lncRNA transcript from the LOC284100 gene located on Chromosome 17q12. LOC284100 is tyrosine 3-monooxygenase/tryptophan 5-monooxygenase activation protein, epsilon polypeptide pseudogene. In addition, lncRNA CEACAMP8 is a 901 bp intragenic lncRNA transcribed from the CEACAMP8 gene located on Chromosome19q13.2. CEACAMP8 is carcinoembryonic antigen-related cell adhesion molecule pseudogene 8. The biological functions of these two LncRNAs remain unclear, although they both have a close relationship with mRNA LPL and lncRNA LOC391533 in the CNC network. LPL plays an important role in hyperlipidemia, and hyperlipidemia is a common complication of preeclampsia. In this study, the qPCR results demonstrated that the three LncRNAs were up-regulated in preeclampsia samples compared with control samples. We proposed that the three lncRNAs may play a role in the development and progression of preeclampsia by interacting with Flt-1 and LPL or contribute to other gene regulation. Additional studies are required to determine how these lncRNAs are involved in the transcriptional and post-transcriptional regulation of related genes in preeclampsia.

 To the best of our knowledge, this is the first study to describe the expression profiles of human lncRNAs in preeclampsia using microarray. We found a collection of deregulated lncRNAs that were aberrantly expressed in the preeclampsia compared to the control group. Most likely, these three deregulated lncRNAs play a key or partial role in the development and progression of preeclampsia. Thus, additional studies are required to determine whether these lncRNAs can serve as new therapeutic targets and diagnostic biomarkers in preeclampsia.

## Materials and Methods

### Patient Samples

 Written informed consent was obtained from all patients, and the study was approved by the Institutional Review Board of Jiangxi Maternal and Child Health Hospital. Forty preeclampsia placenta patients and forty control patients who received a caesarean section were included in the study. Of these patients, six with preeclampsia and five matched controls were used for microarray analysis of lncRNAs, and all patients were used for an extra evaluation. Preeclampsia was clinically diagnosed. The placenta tissue from each subject was snap-frozen in liquid nitrogen immediately after resection. Detailed information of all cases in the study is summarized in Table S2.

### RNA Extraction

 Total RNA was extracted from eighty snap-frozen placenta tissue using TRIzol reagent (Invitrogen, Carlsbad, CA, USA) according to the manufacturer’s protocol. The RNA integrity was evaluated using the Nano Drop ND-1000 spectrophotometer.

### Microarray and Computational Analysis

 RNA purified from total RNA after the removal of rRNA was amplified and transcribed into fluorescent cRNA along the entire length of the transcripts without 3’ bias using a random priming method. The cDNA was labeled and hybridized to the Human LncRNA Array v2.0 (8×60 K, Arraystar). In addition, 33,045 LncRNAs and 30,215 coding transcripts collected from the most authoritative databases, such as RefSeq, UCSC Knowngenes, Ensembl and many related literatures, were detected using microarray. The criteria were as follows: fold-change cut-off: 1.5, fold-change: positive value indicates up-regulation and negative value indicates down-regulation. Log fold-change means a log_2_ value of the absolute fold-change. The fold-change and p-value are calculated from the normalized expression. Arraystar LncRNA Array Protocol: Step 1, Prepare the RNA Sample, kit and reagents: TRIzol Reagent (Invitrogen life technologies), Biopulverizer (biospec), and Mini-Bead-Beater-16(biospec); Step 2, Total RNA Clean-up and RNA QC; Step 3, Prepare labeling reaction; Step 4, Purify the labeled/amplified RNA and labeled cRNA QC; Step 5, Hybridization; Step 6, Microarray Wash; Step 7, Scanning; and Step 8, Extract data using Agilent Feature Extraction Software. The arrays were scanned using the Agilent Scanner G2505B, and the acquired array images were analyzed using Agilent Feature Extraction software (version 11.0.1.1). Quantile normalization and subsequent data processing were performed using the GeneSpring GX v11.5.1 software package (Agilent Technologies). The microarray work was performed by Kang Chen Bio-tech, Shanghai P.R. China. The microarray data discussed in this paper have been deposited in NCBI Gene Expression Omnibus and are accessible with the GEO Series accession number GSE50783 (http://www.ncbi.nlm.nih.gov/geo/query/acc.cgi?acc=GSE50783).

### Construction of the Coding-non-coding Gene Coexpression Network

 The network construction procedures included: (i) preprocessed data: the same coding gene with different transcripts of the median value represent the gene expression values, without specific treatment of the lncRNA expression value; (ii) screen data: remove the subset of data according to the lists that show the differential expression of lncRNA and mRNA; (iii) calculate the Pearson correlation coefficient and use the R-value to calculate the correlation coefficient of PCC between the lncRNA coding genes; and (iv) screen using Pearson correlation coefficient, which was selected when PCC≥0.99 as meaningful and draw the NCN network using cytoscape.

 The circular nodes represent the mRNA, V, on behalf of the lncRNA. The solid lines represent a positive correlation, and the dashed lines indicate a negative correlation. The same color nodes represent co-expressed genes with a similar capacity chart. The node size indicates that the gene expression, where nodes with more expressed gene co-expression have a more extensive relationship with the gene. The K-core indicates the gene expression. Detail information is presented in Figure 1.

### Q-PCR and Statistical Methods

 Total RNA was extracted from frozen placenta specimens using TRIzol reagent (Invitrogen Life Technologies) and then reverse transcribed using the Fermentas RT reagent Kit (Perfect Real Time) according to the manufacturer’s instructions. LncRNA expression in placenta tissues was measured using qPCR and SYBR Premix Ex Taq on the MX3000 instrument. The primers used in this study are shown in Table S9. Three significantly deregulated lncRNAs (LOC391533, LOC284100, CEACAMP8) were evaluated in all of the patients included in this study. Two ug of total RNA was converted into cDNA according to the manufacturer’s protocol. PCR was performed in a total reaction volume of 25 ul, including 10 ul SYBR Premix Ex Taq (2x), 1 ul of PCR Forward Primer (10 uM), 1 ul of PCR Reverse Primer (10 uM), 0.5 ul ROX Reference Dye II (50×)*3, 2 ul of cDNA, and 8 ul of double-distilled water. The quantitative real-time PCR reaction was performed with an initial denaturation step of 10 min at 95°C; and 40 cycles of 95°C (5 seconds), 63°C (30 seconds), 72°C (30 seconds) with a final extension step at 72°C for 5 min. All experiments were performed in triplicate. All samples were normalized to GAPDH. The median in each triplicate was used to calculate the relative lncRNAs concentrations (Ct =Ct median lncRNAs-Ct median GAPDH). The expression fold changes were calculated using the 2-Ct method [17]. The lncRNA expression differences between the preeclampsia and control samples were analyzed using Student’s t-test using SPSS (Version 16.0 SPSS Inc.). A value of p<0.05 was considered statistically significant.

## Supporting Information

Table S1 **Differentially Expressed LncRNAs.**
(XLS)

Table S2 **Basic Medical Records of preeclampsia and control cases.**
(XLS)

Table S3 **LncRNAs Expression Profiling Data.**
(RAR)

Table S4 **GO and Pathway analysis of differentially Expressed LncRNAs.**
(XLS)

Table S5 **mRNAs Expression Profiling Data.**
(RAR)

Table S6 **Differentially Expressed mRNAs.**
(XLS)

Table S7 **GO analysis of differentially Expressed mRNAs.**
(XLS)

Table S8 **Delta-Ct values of Real-Time qPCR in 80 cases and p value.**
(XLS)

Table S9 **Primer Catalog.** We thank all of the participants in this program, all our colleagues who contributed to the generation of the placenta tissue at Jiangxi Maternal and Child Health Hospital, Nanchang Medical College and all our collaborators at the microarray service at KangChen Bio-technology Company in Shanghai.
(XLS)
